# Digital Health Training Programs for Medical Students: Scoping Review

**DOI:** 10.2196/28275

**Published:** 2021-07-21

**Authors:** Lorainne Tudor Car, Bhone Myint Kyaw, Rishi S Nannan Panday, Rianne van der Kleij, Niels Chavannes, Azeem Majeed, Josip Car

**Affiliations:** 1 Family Medicine and Primary Care Lee Kong Chian School of Medicine Nanyang Technological University Singapore Singapore; 2 Department of Primary Care and Public Health School of Public Health Imperial College London London United Kingdom; 3 Centre for Population Health Sciences Lee Kong Chian School of Medicine Nanyang Technological University Singapore Singapore; 4 Department of Public Health and Primary Care Leiden University Medical Centre Leiden Netherlands

**Keywords:** digital health, education, eHealth, medical students, scoping review, electronic health records, computer literacy

## Abstract

**Background:**

Medical schools worldwide are accelerating the introduction of digital health courses into their curricula. The COVID-19 pandemic has contributed to this swift and widespread transition to digital health and education. However, the need for digital health competencies goes beyond the COVID-19 pandemic because they are becoming essential for the delivery of effective, efficient, and safe care.

**Objective:**

This review aims to collate and analyze studies evaluating digital health education for medical students to inform the development of future courses and identify areas where curricula may need to be strengthened.

**Methods:**

We carried out a scoping review by following the guidance of the Joanna Briggs Institute, and the results were reported in accordance with the PRISMA-ScR (Preferred Reporting Items for Systematic Reviews and Meta-Analyses Extension for Scoping Reviews) guidelines. We searched 6 major bibliographic databases and gray literature sources for articles published between January 2000 and November 2019. Two authors independently screened the retrieved citations and extracted the data from the included studies. Discrepancies were resolved by consensus discussions between the authors. The findings were analyzed using thematic analysis and presented narratively.

**Results:**

A total of 34 studies focusing on different digital courses were included in this review. Most of the studies (22/34, 65%) were published between 2010 and 2019 and originated in the United States (20/34, 59%). The reported digital health courses were mostly elective (20/34, 59%), were integrated into the existing curriculum (24/34, 71%), and focused mainly on medical informatics (17/34, 50%). Most of the courses targeted medical students from the first to third year (17/34, 50%), and the duration of the courses ranged from 1 hour to 3 academic years. Most of the studies (22/34, 65%) reported the use of blended education. A few of the studies (6/34, 18%) delivered courses entirely digitally by using online modules, offline learning, massive open online courses, and virtual patient simulations. The reported courses used various assessment approaches such as paper-based assessments, in-person observations, and online assessments. Most of the studies (30/34, 88%) evaluated courses mostly by using an uncontrolled before-and-after design and generally reported improvements in students’ learning outcomes.

**Conclusions:**

Digital health courses reported in literature are mostly elective, focus on a single area of digital health, and lack robust evaluation. They have diverse delivery, development, and assessment approaches. There is an urgent need for high-quality studies that evaluate digital health education.

## Introduction

Digital health (defined as the use of digital technologies for health and health care) is, because of COVID-19, at the center of the pandemic response and support of patients [1,2]. It is a vast and growing field that encompasses the use of digital technology for monitoring, tracking, and informing health; supporting communication among various stakeholders; and managing health data [3,4]. The adoption of digital technologies in health care has increased in recent decades [5,6]. The use of digital technology in health care can reduce errors and costs, increase productivity and efficiency, support clinicians in health care delivery, and allow shared decision-making and self-advocacy for patients [7-9].

There is a pressing need for future clinicians to develop digital health competencies [10,11], and medical schools worldwide have started to introduce digital health education in their curricula [10]. There have been strong pushes for health care systems and services to be digitally enhanced and transformed both in the United States and internationally [12,13]. Patients expect health care providers to offer digital tools as part of health care service delivery [14]. In addition, digital health is a rapidly evolving field in which the new technologies are being developed and emerging, such as artificial intelligence, robotics, wearable devices, and virtual or augmented reality [15,16]. Doctors are expected to keep up with these changes. Correspondingly, a growing number of frameworks outlining digital health competencies for clinicians at various stages of their careers have been developed [4,17-20]. However, health care providers and students have reported a lack of digital health competencies and the need for more digital health–related training [21,22].

Currently, digital health courses are not formally provided or incorporated in most medical school curricula [21]. An analysis of existing studies on digital health courses for medical students should be of use to curriculum planners, educators, and policy makers in the design, development, and adoption of such courses [23]. Therefore, an analysis of existing digital health courses is urgently needed. Such an analysis should explore the content, duration, pedagogy, learning objectives, course integration, assessment methods, format, delivery, and evaluation of reported digital health courses with the aim of informing the development of future courses. Several reviews have been published focusing on training in specific areas within digital health, such as telemedicine [24-26], electronic health record (EHR) training [27], computer literacy, and medical informatics [28,29]. However, digital health education should be comprehensive and systematic [30,31]. To address this gap, we collated and analyzed studies reporting on digital health courses for medical students. Our aim is to inform the development of future courses and identify evidence gaps related to (1) currently available digital health courses for medical students; (2) course design, development, and delivery processes; (3) learning objectives and how they are assessed; (4) use of digital health competency framework and learning theories used during course development; and (5) learning outcomes associated with digital health courses. On the basis of the findings of this review, we aim to provide up-to-date evidence-based recommendations related to digital health courses for future researchers, curriculum designers, and educational policy makers.

## Methods

### Overview

We conducted a scoping literature review following the methodological guidance of the Joanna Briggs Institute [32]. The results were reported in accordance with the PRISMA-ScR (Preferred Reporting Items for Systematic Reviews and Meta-Analyses Extension for Scoping Reviews) guidelines [33]. A search strategy aligned with our aim was developed based on the Joanna Briggs Institute guidelines. The search was performed on November 8, 2019. We searched 6 bibliographic databases indexing biomedical and education journals: MEDLINE, Embase, CINAHL, Education Resources Information Center database (ERIC), PsycINFO, and the Cochrane Library. The search strategy was developed collaboratively and iteratively by the reviewers with support from a medical librarian (Multimedia Appendix 1). For unpublished studies in this area, we searched OpenGrey, ResearchGate, Google Scholar, the first 10 pages of Google results, websites of relevant professional associations (eg, the International Medical Informatics Association and European Federation of Medical Informatics), accreditation councils (eg, the US Accreditation Council for Graduate Medical Education), key government websites, and other organizations with the mandate of training and lifelong learning of health care professionals. We also screened the reference lists of the included studies based on the eligibility criteria.

### Eligibility Criteria

We included all articles published between January 1, 2000, and November 6, 2019, because digital health is a rapidly evolving area and has changed substantially over the last 20 years. We included articles published in English and assessed their eligibility. The inclusion criteria were developed in alignment with the aims of our review (Multimedia Appendix 2). We defined *digital health* as any form of information technology (IT) used in health care practices or health professions education. For a list of technologies classified as digital, please refer to Multimedia Appendix 2. We included all types of primary studies on digital health, clinical, or health informatics training at all medical schools, regardless of setting. We included experimental (eg, randomized controlled trials [RCTs] and before-and-after studies), observational (eg, cohort studies), and descriptive (eg, case studies and qualitative studies) studies. We included both controlled experimental studies (ie, studies in which digital health education was compared with another intervention or no intervention at all) and uncontrolled ones (ie, studies that examined only 1 group of participants receiving digital health training). We also included quasi-RCTs, that is, RCTs in which participants were allocated to different arms of the study without a proper randomization method.

### Screening and Data Extraction

We screened the articles by applying our predefined inclusion and exclusion criteria first to the title and abstract and then to the full texts of the relevant articles. For the title and abstract screening, we screened the articles independently in pairs by using Covidence (Veritas Health Innovation Ltd) [34]. Any discrepancies or disagreements between the reviewers were resolved through discussion and consensus, and when required, a third reviewer was engaged as an arbiter. For full-text screening, the same screening process was followed by using EndNote X8 (Clarivate) [35]. The data extraction form was aligned with the research questions or objectives (Multimedia Appendix 3). Two review authors extracted the data independently and discussed them until they reached a consensus on the final extracted data.

### Data Synthesis

We analyzed the identified digital courses in terms of year or type of study, digital health topic, format of the course, development, delivery, and assessment approaches. We then narratively synthesized the contents of the identified digital health courses in each area, including learning objectives and the associated challenges related to the development and implementation of digital health courses for medical students. We classified the digital health courses into different domains according to the terminology and aims presented in the included studies. For example, studies focusing on EHR or medical informatics training were classified under the EHR or medical informatics domains, respectively. As medical informatics encompassed diverse digital health topics in the included studies, we identified and presented the specific medical informatics that the courses focused on.

## Results

### Study Characteristics

**Figure 1:**
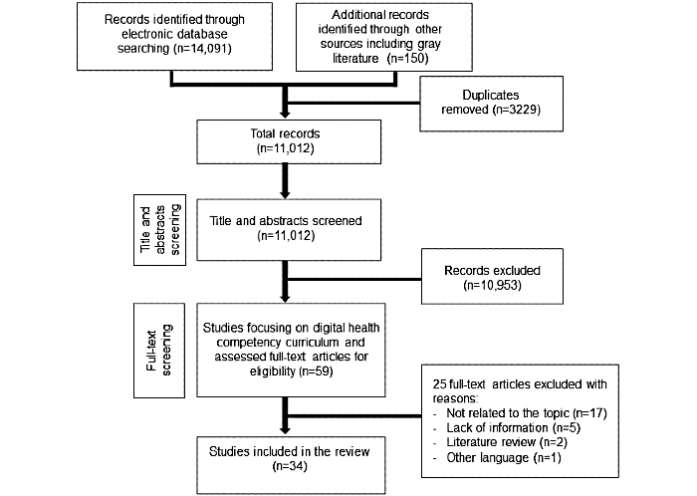
PRISMA (Preferred Reporting Items for Systematic Reviews and Meta-Analyses) flowchart for scoping reviews on digital health courses for medical students.

The search strategy yielded 14,241 publications, and of these, 14,091 (98.95%) were from database searches and 150 (1.05%) were from gray literature. In total, 34 articles met the inclusion criteria (Figure 1). Most of the studies (22/34, 65%) were published between 2010 and 2019 and were uncontrolled before-and-after studies (24/34, 71%). Other study designs reported in the included studies were case studies (5/34, 15%) [4,36-39], controlled before-and-after studies (4/34, 12%) [19,40-42], and a quasi-RCT (1/34, 3%) [43].

### Digital Health Courses’ Scope, Students, and Settings

Of the 34 included studies, 17 (50%) focused on medical informatics [4,18,37,39,40,42-53], 8 (24%) on EHR skills [3,19,41,54-58], 3 (9%) on computer literacy [59-61], 3 (9%) on telemedicine [36,62,63], 2 (6%) on basic programming [38,64], and 1 (3%) on mobile health (mHealth) [65]. Most of the studies (20/34, 59%) were conducted in the United States [3,18,19,39,41,44-47,51-58,60,62,63,66]. The remaining studies were conducted in Australia [37,64], France [40], Germany [48,49], Hungary [59], Canada [38], Croatia [4], Commonwealth of Dominica [50], Taiwan [61], the United Kingdom [42], the Philippines [37], and Romania [43].

### Course Structure and Content

Most of the digital health courses (24/34, 71%) were integrated into existing courses [3,4,18,19,37,39-44,46-49,51-56,58,59,65], and only a few courses were reported as stand-alone courses (7/34, 21%) [36,38,45,57,61-63]. Similarly, most of the digital health courses were provided as elective (18/34, 53%) [3,18,36,37,39,42,45,48-52,55,59,60,62,63,65], and only a few courses were provided as mandatory courses (7/34, 21%) [40,43,44,46,53,54,56]. Of the 34 courses, 2 (6%) biomedical informatics courses were offered as both elective and mandatory depending on the year of the study [4,47].

The included studies evaluating medical informatics courses (17/34, 50%) focused on different areas of medical informatics, such as the use of a clinical decision support system, data privacy and security, medical image processing, biosignal analysis, basics of electronic medical records, patient management systems, basics of IT in medicine, community health information tracking systems, data management (eg, data storage and retrieval), information literacy (eg, formulating clinical questions, searching online bibliographic databases, and searching evidence-based resources), and communication technology [4,18,37,39,40,42-53]. The duration of the medical informatics courses ranged from a minimum of 1 session lasting 3.5 hours [44] to regular training over 3 years [39] (Multimedia Appendix 4 [3,4,18,19,36-65]).

Of the 34 included studies, 8 (24%) reported courses on EHR and mainly focused on knowledge and skills related to EHR use for first- to fourth-year medical students [3,19,41,54-58]. The courses focused on the general application of EHR in clinical settings lasting from 1 hour [41] to throughout the preclinical years of medical school [54]. Lee et al [41] reported a 1-hour lecture on patient-centered EHR use for second- and third-year students, and the course was integrated into the clinical skills course. Milano et al [56] reported a 2-week EHR course for first- and third-year medical students, which was incorporated into a third-year family medicine clerkship hands-on course, working on a simulated EHR using virtual patient simulation. Connors et al [19] presented an EHR course for first- to third-year medical students, in which the course materials, including laboratory and pathology reports, were provided as a case-based EHR course to familiarize the students with EHR skills. Wagner et al [54] presented an EHR course for medical students during their preclinical years of training and focused mainly on content associated with online health record submission tools for an EHR system. Ferenchick et al [3] also presented a short stand-alone online EHR course on the meaningful use of electronic clinical data for disease management and outcomes. The online course consisted of 15 online tutorials on applications of EHR and lasted 71 minutes in total. Gomes et al [57] presented a stand-alone EHR online video course for medical students through a blackboard platform, which mainly included a narrative video of PowerPoint (Microsoft Corporation) presentations on different functions of EHR and its applications. The remaining studies (2/8, 25%) focused on EHR courses for both third- and fourth-year medical students, and the courses covered topics on the overview of EHR, order entry, patient information review, chart documentation [58], and EHR-based patient communication skills [55].

Of the 34 included studies, 3 (9%) focused on computer literacy courses for medical students focusing on basic computer applications and skills in clinical practice, the use of social media tools for self-learning, and digital game-based learning in medical education [59-61]. The duration of the courses varied from 3 weeks [60] to 17 weeks [61]. Wan et al [61] reported a stand-alone entry-level elective course on basic computer concepts for medical students, in which the students are expected to spend 2 hours per week for 17 weeks in self-learning, cooperative learning from a book club, and game-based learning from online Jeopardy-like games. Similarly, Gibson and Silverberg [60] reported an elective computer literacy course that lasts for 3 weeks, in which the students receive 7 hours of hands-on training on computer literacy, followed by a test. Mesko et al [59] presented a 12-week digital or computer literacy course for medical students using social media tools and gamification approaches.

Of the 34 included studies, 2 (6%) reported a computer programming course for medical students [38,64]. Law et al [38] described a 14-month stand-alone elective computer programming course for medical students, which consists of introductory sessions (3-4 sessions depending on skill level) for the first 3 months and 11 sessions over a 11-month period. Liaw and Marty [64] presented a basic programming course consisting of software use, didactic workshops, and conversations (Multimedia Appendix 4).

Of the 34 included studies, 3 (9%) reported a telemedicine elective course for second- to fourth-year medical students [36,62,63]. The courses focused on the introduction of telehealth and telemedicine, lasting from 9 hours [63] to 1 full semester [36]. Of these 3 studies, 2 (67%) reported a single-semester elective course on mHealth [65] or telemedicine [36], and 1 (33%) reported a 1-month biomedical informatics course for first- to fourth-year medical students [47]. The biomedical informatics course was a compulsory core module course for first- to third-year medical students and an elective module for fourth-year medical students.

### Delivery Approaches

Most of the courses (22/34, 65%) used a blended format of delivery (ie, a combination of online module or offline learning [eg, computer-based spreadsheet and presentation software packages, PowerPoint presentation, CD-ROM, or DVD] and traditional approaches such as small group discussions, lectures, and classroom interactions) [18,36,38,40-44,47,49-55,59-61, 63-65]. Learning content was delivered in full online mode in a few courses (7/34, 21%) [3,37,45,46,57,58,62]. Of these 7 courses, 2 (29%) were delivered as massive open online courses through a learning management system [37,45], 2 (29%) used mixed modalities of both online and offline learning [4,39], and 1 (14%) focused on stand-alone EHR simulation in offline mode [19], whereas virtual patient simulations were used in 2 (29%) courses (Multimedia Appendix 5) [48,56].

### Educators Involved in Digital Health Courses

Of the 34 included studies, 14 (41%) reported on the trainers or educators involved in the development and delivery of digital health courses [18,38,39,41,44,45,50,51,53,54,56,57,63,65]. The educators mentioned in the included studies were mostly medical librarians and faculty members, including clinicians. Of these 14 studies, 7 (50%) reported the involvement of other staff in the digital health courses such as IT support teams [63], patients [41], patient educators [51], and student assistants [45,54,63,65], whereas 4 (29%) mentioned the required skills or training for the staff members developing or delivering digital health courses [38,54,63,65].

### Digital Health Course Development

Of the 34 included studies, 17 (50%) reported course development processes, including expert consultations, piloting of the course, literature review, and review of other programs in the course development [4,18,37,41,43-45,47,49, 51,53,56-59,63,65]. Expert consultations used in the studies included seeking feedback from the EHR vendors, librarians, faculty members, clinicians, and researchers [18,37,41,44,45,47,51,53,56-58,63,65]. Of these 17 studies, 4 (24%) [18,45,51,56] used a literature review and expert consultations for the development of courses, 2 (14%) reported piloting of the course before being incorporated into a medical program [51,56], 7 (50%) used expert consultations alone [37,41,53,57,58,63,65], and 2 (14%) carried out a literature review only to design the course [4,59]. Of the 17 studies, 3 (21%) studies piloted the course with expert consultation [47], without expert consultation [43], or only after literature and curriculum review [49], whereas 1 (6%) study used both curriculum review and expert consultation methods [44].

### Digital Health Courses’ Learning Objectives

Learning objectives were presented as general or specific depending on the topics of the digital health courses. General learning objectives were mainly related to the improvement of medical students’ medical informatics knowledge, skills, and attitudes. Specific learning objectives were presented as competencies related to a particular clinical or preclinical setting and focused on a specific aspect of the use of digital health technology in health care. The details of the learning objectives presented in each digital health course are presented in Multimedia Appendix 6 [3,4,18,19,36-65].

Of the 34 included studies, 11 (32%) reported the developmental steps for learning objectives, such as evaluation of other available digital health courses; inputs from content experts and faculty members; and following specified protocols, steps, or guidelines to develop learning objectives for the presented courses [18,39,44,45,47,51,53,56,58,60,65]. The remaining studies did not follow any specific guidelines or protocols to develop learning objectives for digital health courses.

### The Use of Digital Health Course Frameworks

There was limited use of digital health competency frameworks in course development. Of the 34 included studies, only 6 (18%) reported that course developers used frameworks or guidelines to develop digital health courses [4,18,19,44,52,58]. Kern and Fister [4] reported that their medical informatics course was based on the International Medical Informatics Association Recommendations on Medical Informatics Education for IT users and adjusted to students’ attitudes toward medical informatics and the position of the courses in the first and fifth year of the medical program. Connors et al [19] reported that the learning objectives of the EHR courses were based on the informatics competencies outlined in the 2001 report of the Institute of Medicine. Of the 6 studies, 3 (50%) developed their learning objectives for medical informatics courses based on the competencies specified in the Association of American Medical Colleges Medical School Objective Project [18,44,52], and 1 (17%) study by Pereira et al [58] followed Kern and Fister 6-step course design framework to develop an EHR course for medical students.

### Assessment and Evaluation of the Digital Health Courses

For the assessment of learning outcomes, the courses used paper-based assessments in the form of surveys, in-person observations (eg, objective structured examinations), and/or online assessment methods (ie, online surveys). Of the 34 digital health courses, 11 (32%) used paper-based assessments [3,4,18,38,41-43,47,52,64,65], 10 (29%) used online assessments [44-46,50,51,54,57,58,60,62], 3 (9%) used in-person observations [18,56,63], and 6 (18%) used both paper- and online assessment methods [36,53,56,59,61,63]. The remaining courses (7/34, 21%) did not assess student outcomes; thus, no assessment methods were reported [19,37,39,40,48,49,55].

Of the 34 included studies, 30 (88%) evaluated digital health courses that mostly used uncontrolled before-and-after design. Changes in learners’ knowledge related to telehealth, EHR, or medical informatics were assessed in one-third (10/30, 33%) of these studies [36,41,45,47,50,51,53,57,58,63]. Of these 10 studies, 5 (50%) reported an improvement in learners’ knowledge related to telehealth [36,63], EHR [41,57], and biomedical informatics [47]. Of the 30 studies, 9 (30%) reported digital health competency skills of the students before and after taking part in the digital health course [3,41,42,55,56,59-61,64], of which 89% (8/9) of studies reported that digital health courses were associated with an improvement in medical students’ digital skills [3,41,42,56,59-61,64].

Of the 34 included studies, 16 (47%) assessed students’ attitudes toward a medical informatics course [4,18,40,42,45,46, 48,49,52], EHR skills [41,54,57], mHealth [65], telemedicine [62], programming [64], and computer literacy courses [59]. Most of the studies reported positive attitudes toward digital health courses. Of these 16 studies, 3 (19%) reported students’ satisfaction with medical informatics [43,52] and telemedicine courses [36], whereas 1 (6%) assessed students’ engagement with learning content and reported that 65% of the students read more than 75% of their learning content [61]. Another study assessed the information-seeking behaviors of students and reported that the students showed a higher degree of use of information resources [44].

### Challenges Related to Course Development and Implementation

Of the 34 included studies, 9 (26%) reported students’ and educators’ challenges related to digital health courses. Most of the reported challenges were associated with course development and implementation [4,43,45,47,48,60,62,64,65]. The challenges faced by students attending digital health courses included incomplete assignment submission owing to errors in the learning management system [45], limited participation rate [62], and a lack of perceived usefulness of the courses as part of preclinical training [43]. From the educators’ perspective, the challenges included the demands for providing timely feedback to students [45], recording and producing lectures for optimum accessibility, mastering online learning tools [45], inadequate cooperation between IT support persons and health care professionals to deliver digital health courses [4], poor computing and typing skills [64], and a lack of clinically trained faculty for content creation and teaching [47]. Other challenges included the inadequacy of technological infrastructure such as software, hardware, IT systems issues [64]; implementation issues (eg, converting paper content to digital format) [64]; and design and development of the course (tailoring of the course content to real-life learning and teaching facilities within a financially constrained context) [65].

## Discussion

### Principal Findings

We found 34 studies that presented digital health courses for medical students. The included studies mostly focused on medical informatics, followed by EHR and telemedicine, and targeted medical students throughout their years of study. Courses were mostly delivered using online and blended approaches and integrated into curricula as elective courses. The duration of the digital health courses in the included studies ranged from a minimum of 1 hour to a maximum of 3 years. Only a few studies reported evaluation data for the courses, and these largely reported improvements in knowledge, skills, attitudes, satisfaction, and students’ engagement with digital health courses. The courses reported in the included studies had a very diverse approach to course development. Only one-third of the included studies followed specified protocols, steps, or guidelines to specify the learning objectives for digital health courses. Similarly, most of the included courses did not refer to the use of a digital health competency framework during course development.

Most of the digital health courses were offered as elective courses. Given the need for a digitally competent health workforce, it is important that digital health courses become part of the core curriculum. In addition, studies focused on one area of digital health, mostly medical informatics, followed by EHR skills and computer literacy. Medical informatics courses within the included studies varied and ranged from the basic concepts of medical informatics, theories, and applications to details about health information management and systems. Many medical informatics courses focused primarily on information literacy and the development of evidence-based medicine skills. It is important to acknowledge the constant progress in digital health and the fact that studies published before 2010 could not have included training on more novel digital health applications such as the use of artificial intelligence or big data. In addition, digital health is a vast and growing field. As such, it may need to be incorporated into the medical curriculum in a stepwise, modular manner, with smaller courses focusing on individual and specific areas. Correspondingly, half of the studies included in our review focused on a particular digital health area. However, it is essential to have a comprehensive overview of all digital health competencies that the curriculum focuses on, and existing digital health competency frameworks may provide a useful guide in the development of courses. However, they were only mentioned in a small number of courses. Future digital health courses should focus on emerging technologies such as virtual consultation, mHealth, smart wearable devices, activity trackers, and other smart monitoring devices.

Most of the included studies were uncontrolled before-and-after studies; evaluated the effectiveness of digital health courses; and reported a number of learning outcomes, including changes in knowledge, skills, and attitudes toward the course. Although the findings related to the reported learning outcomes from the studies were in favor of a digital health course, there is a need for more robust evaluations of the effects that digital health courses have on learning outcomes, which was also highlighted in recent studies focusing on telemedicine [26,31,67] and clinical informatics courses [28,29]. Currently, there is only limited evidence, and more evaluation and implementation research is recommended.

Our review has several strengths, including the comprehensiveness of the search, covering major bibliographic databases; robust screening; data extraction; and data analysis. However, because this is a novel area of research, there may be some reports of digital health courses in gray literature that we may have missed. In addition, we included studies published from 2000 onward, and we may have missed studies published before 2000. However, because of recent advances in digital technologies within the last two decades, we decided to focus on the most relevant studies on the topic. Finally, the description of the design and implementation of digital health courses (eg, specific learning objectives or assessment approaches) in some studies was limited, precluding a more in-depth analysis and presentation of the findings.

### Recommendation for Implementation and Further Research

To the best of our knowledge, this is the first attempt to comprehensively review studies evaluating digital health topics–related courses for medical students. One recently published study looks at medical students’ training in eHealth from 2014 onward and lacks information related to curriculum design, developments, and assessments [68]. We identified several gaps related to digital health courses, such as the need for standardization of course design and development, course integration, assessment methods, studies from different settings, and evidence on the effectiveness of various course formats.

Most of the included studies focused on medical informatics courses. More research is needed on other areas of digital health, such as mHealth and telemedicine. In addition, most of the included studies were from high-income countries. There is a need for context-specific studies in diverse settings, including low- and middle-income countries. High heterogeneity in reporting in the included studies highlighted the need for standardized reporting guidelines and validated outcome assessment tools. Finally, more high-quality studies assessing the effectiveness of different forms of digital delivery approaches in improving digital health–related learning outcomes for medical students are needed because most of the included studies are uncontrolled before-and-after studies or case studies.

### Conclusions

Current digital health courses for medical students that have been evaluated or reported in the literature are mostly elective and showcase diverse delivery, development, assessment, and evaluation methods. The limited evaluation data show improvement in students’ knowledge, skills, and attitude toward digital health course outcomes. The COVID-19 pandemic has increased the importance of digital health, with a substantial increase in the use of remote consultation models and greater use of electronic prescribing [69]. Doctors and other health professionals need to be adequately trained to work in this new environment, where a greater proportion of health care is delivered by digital methods. Hence, further high-quality studies assessing the effectiveness of digital health courses on students’ learning outcomes are needed. There is also a need for standardization and development of guidance specifying different digital health areas, terminology, learning objectives, optimal development and delivery approach, duration, assessment method, and structure of the courses.

## Appendix

Multimedia Appendix 1 MEDLINE search strategy and keywords used for searching gray literature.

Multimedia Appendix 2 Inclusion and exclusion criteria.

Multimedia Appendix 3 Data extraction form.

Multimedia Appendix 4 Characteristics of the included studies on digital health training for medical students.

Multimedia Appendix 5 Digital health courses’ delivery approaches.

Multimedia Appendix 6 Digital health course development process and learning objectives.

## Abbreviations

EHR: electronic health record
IT: information technology
mHealth: mobile health
PRISMA-ScR: Preferred Reporting Items for Systematic Reviews and Meta-Analyses Extension for Scoping Reviews
RCT: randomized controlled trial
