# Supplementary material for: Digital Health Training Programs for Medical Students: Scoping Review
Source: JMIR Med Educ. 2021 Jul 21;7(3):e28275. doi: 10.2196/28275 (PMC8339984; doi:10.2196/28275)
Supplement: Multimedia Appendix 1 [file mededu_v7i3e28275_app1.docx]

## Multimedia Appendix 1:

## Search strategy for Medline

1 Computer User Training/ or Computer Literacy/ (3328)

2 ((ehealth or e-health or digital health or health technolog* or health information technolog* or informatics or educational technolog* or electronic medical record? or electronic health record? or electronic patient record? or digital health record? or electronic prescribing or eprescribing or telehealth or mhealth or m-health or telemedicine or telemonitoring or clinical decision support system? or teleradiology or telenursing or hospital information system? or computer assisted decision making) adj4 (competenc* or literac* or skill? or capabilit* or capacit* or readiness or curricul*)).ab,ti. (1438)

3 exp Health Personnel/ or exp Students, Health Occupations/ (547308)

4 (health personnel or healthcare profession* or health profession* or healthcare worker? or health worker? or healthcare provider? or health provider? or health service provider? or physician? or doctor? or medic* or premedic? or medical student? or nursing student? or nurse student? or medical graduate? or undergraduate medical or postgraduate medical or health graduate? or resident? or medical practitioner? or general practitioner? or general medicine or family medicine or primary care or nurs* or dentist? or dental or predent* or pharmac*).ab,ti. (3464534)

5 3 or 4 (3677803)

6 2 and 5 (988)

7 1 and 5 (1980)

8 6 or 7 (2861)

9 limit 8 to yr="1990 -Current" (2748)

(Time limit: 1^st^ January 1990 – 6^th^ November 2019)

Number of search results from Medline using this search strategy: 2,748

**Total number of search results across the 6 databases: 14,091**

## Keywords used for searching the grey literature

1. Digital health competency
2. e-health competency
3. e-health literacy
4. eHealth literacy
5. ehealth competency
6. eHealth competency
7. ehealth literacy
8. Digital health literacy
9. Computer literacy
10. Computer user training
11. Health informatics competency
12. Information technology competency
13. Informatics literacy
14. Informatics competency
15. Information literacy need
16. Information literacy skill
17. Biomedical competency
18. Digital health capability
19. e-health capability
20. Health technology competency
21. Health information technology
22. Digital health skill
23. Digital health capacity
24. e-health skill
25. ehealth skill
26. eHealth skill
27. e-health ready
28. ehealth ready
29. eHealth ready
30. Digital health ready
31. Health informatics
32. Clinical informatics
33. Bioinformatics
34. Medical informatics
35. Imaging informatics
36. Biomedical informatics
37. Surgical informatics
38. Clinical competence
39. Competency-based education
