# Supplementary material for: Digital Health Training Programs for Medical Students: Scoping Review
Source: JMIR Med Educ. 2021 Jul 21;7(3):e28275. doi: 10.2196/28275 (PMC8339984; doi:10.2196/28275)
Supplement: Multimedia Appendix 2 [file mededu_v7i3e28275_app2.docx]

**Multimedia Appendix 2.** Inclusion and exclusion criteria.

**Inclusion criteria**

- Relevant articles published in English, from peer-reviewed journals as well as grey literature sources so long as they cover courses on digital health competencies for medical students’ education (including all stages of the course design process - needs assessment, learning objectives, content, teaching methods, assessment and course evaluation)
- Studies focusing on medical students and the digital health courses at medical schools - including course development, implementation and evaluation. We include all stages of the course design process - needs assessment, learning objectives, content, teaching methods, assessment and course evaluation.
- All pre-service or undergraduate medical students enrolling in medical schools.
- ehealth or e-health or digital health or health technology or health information technology, health informatics, educational technology, electronic medical record (EMR), electronic health record (EHR), electronic patient record, digital health record, email/database/electronic health, telehealth, mhealth, m-health, smartphone applications (apps), telemedicine, telemonitoring, teleradiography or anything that starts with “tele”, computer-assisted or computer-based (i.e. computer-assisted history taking), computerized disease registries, clinical decision support, computerized provider/physician order entry, electronic prescribing (e-prescribing), artificial intelligence (AI), clinical management systems, hospital information system, internet (online), intranet, LAN, web, simulated/simulation learning modality of a virtual or electronic nature, virtual reality/anything involving distance learning, use of “software name”, robot/robotic network, remote, computer-based, CD-ROM, DVD, telemedicine/teleadvice for learning, “streaming”, gaming, gamification, MOOC, digital games/anything, m or mobile device, multimedia, 2-D, 3-D, podcast, video/videotape, ”serious games” as part of digital games, head mounted display/goggles, immersion/immersive experience as part of virtual reality, avatar, computer generated patient, augmented reality, mixed reality, LapSim, Box trainer, 2^nd^ life, general or non-specific games/interactive/training program/workshop with no mention of any “e” or “digital” component.

**Exclusion criteria**

- Articles published before 1^st^ January, 2000
- Digital Health courses focusing on non-digital health competencies for both pre- and in-service health professionals
- Competencies related to evidence-based practice and information literacy, except when other digital health competencies are also covered within the course
- Digital health courses for other populations (i.e. patients, general population, students, trainees and professionals in professions other than health, trainers/educators with both health and non-health backgrounds, staff/students in “traditional medicine,” alternative medicine“, “complementary medicine”, engineering, Chinese medicine, homeopathy, acupuncturist, herbalist, Ayurvedic, chiropractor, students in basic science including pharmacology, anatomy, naturopath, non-medical/clinical social worker or psychologist (except clinical psychologist) or technologist with no medical/clinical or related term in front.
- Non-digital technology/concepts (i.e. physical/real tools, mannequin without digital components, plastic dummy with haptic feedback but does not include “e” or “digital”, hard copies, cassettes, real/live patients used as educational tools, blended learning where all learning components in the intervention are clearly NOT “e” or “digital”, standardized patients using real patients)
- Literature with descriptions of digital health policies/initiatives at any level (institutional/regional/national/global) unless a digital health course has been developed and discussed
- Literature covering assessment of the digital health competencies of pre- and in- service health professionals (with or without training)
- Literature on the acceptability, usage, preferences, perceptions, attitudes, barriers and facilitators related to any digital technology including but not limited to: (a) computers, digital health tools/health information technology/telemedicine equipment/software (b) technology for training/curriculum/courses/interventions
- Literature on the applications of digital technologies in health, technology related trends in health; as well as the effect of digital health on healthcare quality, patient safety, etc.
- Literature on standards related digital health infrastructure, IT sophistication/capability/capacity of health systems/organizations or technical descriptions/modifications/development of software in the healthcare contexts (e.g. software changes to adapt it to workflow, improve usability)
- Opinion pieces/commentaries/viewpoint articles/editorials/letter to Editor
- Conference abstracts, posters and book reviews
