# Supplementary material for: Digital Health Training Programs for Medical Students: Scoping Review
Source: JMIR Med Educ. 2021 Jul 21;7(3):e28275. doi: 10.2196/28275 (PMC8339984; doi:10.2196/28275)
Supplement: Multimedia Appendix 4 [file mededu_v7i3e28275_app4.docx]

Multimedia Appendix 4: Characteristics of the included studies on digital health training for medical students

| **Study ID (first author and year of publication), Country, Study design** | **Participants, Institution(s)** | **Digital health area and course content** | **Modality (offline course, online course, blended course); Type of course (elective, core, integrated, standalone)** | **Duration and structure of the course** | **Course Development approach and frameworks used (Steps involved)** | **Assessment methods** | **Evaluation of the course** |
| --- | --- | --- | --- | --- | --- | --- | --- |
| Behrends 2011 [53], Germany, Uncontrolled before and after study | Third year medical students, Hannover Medical School | ***Medical informatics:***  The course focused on basics of medical informatics, i.e. clinical decision support systems, medical information systems, healthcare telematics, data privacy protection, health technologies, medical image processing and biosignal analysis as well as general aspects of using the internet in medicine. | Online course (Virtual Patient Simulations);  Elective course, Integrated into third year of medical program | Duration: 23 hours of lectures and tutorials over 3 weeks. | Course Development approach: NA  Frameworks used: NA | Survey | **Attitude** towards medical informatics course (before and after the course): no difference |
| Blumenthal 2005 [18], USA, Uncontrolled before and after study | First- and third-year medical students, Georgetown University School of Medicine | ***Medical informatics* - *Preclinical Informatics*:**  The instructional intervention consisted of large group sessions in the library’s computer classroom, facilitated by a faculty-librarian team, or individual consultations with a librarian.  ***Clinical Informatics:***  The workshops were highly interactive and unstructured, with an emphasis on hands-on practice time and group discussion on formulation of clinical questions, critically appraise the literature, and applications of their findings to patient scenarios. | Blended Online Course  (hands-on workshop, group discussions and online activities);  Elective course, Integrated into third year of medical program | Duration: 1 week (workshops were held in the second week of the four-week family medicine clerkship in Year 3). | Course Development approach: Literature Review, Expert Consultation  Frameworks used: 1) Association of American Medical Colleges (AAMC) Medical School Objectives Project  2) Accreditation Council for Graduate Medical Education (ACGME) Outcomes Project | In person observations, Rating | **Attitude** towards usefulness of clinical informatic course: Overall rating of 4.3 out of 5-point scale (1=not useful, 5=extremely useful) |
| Breil 2010 [54], Germany, Uncontrolled before and after study | medical students (Unspecified) and informatic students, Department of Medical Informatics and Biomathematics, University of Münster | ***Medical Informatics:***  The course included case studies on medical informatics and additional multidisciplinary course on “Health Care Informatic System”, which covered the different sub-topics such as project management, system analysis, system selection, system implementation and project conclusion. | Blended Offline Course (lectures and computer-based activities);  Elective course: integrated into medical curriculum. | Duration: 16 hours classroom teaching plus comprehensive project documentation and implementation tasks for the informatics students. The course is designed for 16 students per  term (eight medical and eight informatics students). There is a workload of 2 Credits in total. | Course Development approach: Literature Review; Evaluation of existing courses/curricula;  Piloting the course  Frameworks used: NA | Rating, free-text comments | **Attitude** towards the course: The average score was between 38.9  and 56.3 out of 100. The students reported positive attitudes towards the course. |
| Brockes 2017 [45], Switzerland, Case study | Medical students (2^nd^, 3^rd^ and 4^th^ year), University of Zurich | ***Telemedicine:***  The course covered both theories and practical sessions including consultation with telemedical doctors and telephone consultation. Additionally, the course includes lectures on e-Health and Sensoring which involves telemonitoring of cardiac patients. | Blended online course (online lectures/homework plus lectures in the classroom) | Duration: One semester  Course structure: Includes lectures and practical sessions on teleconsultations | Course Development approach: NA  Frameworks used: NA | Ratings, Students’ feedback | 1) **Satisfaction**: improved throughout the years [mean scores: 4.71 +/-0.74 (in 2009), 4.86 +/– 0.93 (in 2011), 5.1+/ – 1.5 (in 2016)]  2) **Knowledge:** understanding of telemedicine as a supplement in traditional medical consultations increased throughout the years [mean scores: 5.02 +/– 0.96 (in 2009), 5.25 +/– 0.81 (in 2011), 5.7 +/– 0.6 (in 2016)] |
| Brown 2003 [25], USA, Uncontrolled before and after study | Medical students (first and second year) | ***Medical Informatics:***  The course covered the topics on information literacy, literature search, critical appraisal of literature, and workshop on evidence-based medicine resources. | Blended Online Course;  Mandatory course, Integrated into medical program | Duration: 3.5 hours  Course structure: A workshop (1.5 hour) is given in the first year, two 1-hour workshops are given in the second year.  The course provided hands-on workshop including online literature search. | Course Development approach: Evaluation of existing courses/curricula; Expert consultation  Frameworks used: NA | Survey (online) | **Information seeking behavior** of students: the students showed a higher degree of usage of information sources |
| Bulik 2010 [64], USA, Uncontrolled before and after study | Fourth year medical students, University of Texas Medical Branch, Galveston, Texas, USA | ***Telemedicine:***  The course covered topics related to basic information about telemedicine and a detailed guide to planning and starting a telemedicine program as well as business aspects in the planning process of beginning a telemedicine program. | Online Course;  Elective, Standalone course | Duration: 4 weeks.  Course structure:  (i) Two online introductory courses to telemedicine.  (ii) Site visits to near and distant telemedicine sites.  (iii) A reflective writing paper. | Course Development approach: NA  Frameworks used: NA | Survey (Online) | **Attitude** towards the course: reported positive attitudes towards usefulness of the course |
| Burgun 2006 [49], France, Controlled before and after study | Medical students (third year) | ***Medical Informatics:***  The course covered basic topics of medical informatics such as search engines, thesaurus vs natural language, database and web pages. | Blended online course (face-to-face tutorial group meetings, after which an online search is conducted);  Mandatory, integrated course | Duration: 6 hours  Course structure:  1) A first tutorial group meeting,  2) Personal work and  3) A second tutorial group meeting | Course Development approach: NA  Frameworks used: NA | Survey | **Attitudes** towards the course: no significant difference between the two groups |
| Burnette 2012 [26], USA, Case study | medical students, unspecified year, University of Illinois at Chicago | ***Medical informatics:***  The course covered different aspects of medical informatics such as clinician’s perspective, health literacy, the research process, teaching and learning, and lifelong learning. | Online Course (delivered by Blackboard - Learning Management System);  Elective: Standalone course | Duration: 2-weeks  Course structure: Asynchronous online course (assignments) delivered via Blackboard. Assignments included posting commentary using discussion board, database customization, syndication creation, bibliographic management and web personalization. | Course Development approach: Literature review; Expert consultation  Frameworks used: NA | Assignments, Survey (online) | **Attitude:** students reported that the information they learned would be useful for residency and future medical practice. The majority reported feeling greater competence and confidence in MI as a result of the course, particularly with regard to the challenges of the electronic medical record (EMR), scholarly communication, information access issues, definition of MI, and description of issues of information storage and retrieval. |
| Connor 2003 [55], Dominica, Case study | Pre-medical students, Ross University School of Medicine | ***Medical informatics:***  The course covered the topics related to different areas of medical informatics such as use of information technology in healthcare, managing large volumes of knowledge, using clinical practice guidelines and digital skills related to communication and problem-solving in healthcare. | Blended Offline Course (hands-on computer session, student journal club, assignments);  Elective course | Duration: NA  Course structure: the 10 sessions including hands-on computer session, journal club and effective searching of medical databases. | Course Development approach: NA  Frameworks used: NA | MCQs (online) | NA |
| Connors 2007 [19], Case study, USA | First to third year medical students, University of Kansas (KU) School of Nursing but rolled out to KU School of Medicine | ***EHR***:  The course consisted of topics on the use of EHR and development of a management plan for patients using computerized physician order entry and e-prescribing functionality used in an outpatient clinic. | Offline Course (Case presentation through EHR Simulation);  Integrated into medical program | Duration: NA  Course structure: the course included EHR based clinical cases studies including documentation of patient history and physical findings as well as developing clinical management plan for patients. | Course Development approach: NA  Frameworks used: Institute of Medicine report 2001 | NA | NA |
| Ferenchick 2013 [3], USA, uncontrolled before and after study | Medical students (third year) | ***EHR:***  The course covered topics related to the use of HER, electronic analysis of data entered into structured field and decision-support tools used in healthcare (e.g., checking drug-drug interactions and electronic information exchange). | Online Course;  Elective course: Integrated into medical program | Duration: 71 minutes  Course structure: 15 online tutorials related to basic EMR functions including MU competencies. And remote access to remotely access EMR playground to complete 10 MU tasks in the chart of simulated patient. | Course Development approach: NA  Frameworks used: NA | OSCE | **Skills:** Students with meaningful use errors demonstrated poorer performance on end-of-clerkship professionalism assessments and lower observed structured clinical examination scores |
| Fernandez-Marcelo 2012 [46], Case study, Philippines | Sophomore/Second year medical students, University of the Philippines) (UP-Philippines) | ***Medical informatics***:  The course described the use of Community Health Information and Tracking System (CHITS) for health information management on TB cases for training medical students. | Online Course (Moodle - Learning Management System);  Elective course, Integrated into medical program | Duration: 4 hours  Course structure: 4-hour session on medical informatics. | Course Development approach: Expert Consultation  Frameworks used: NA | NA | NA |
| Fernando 2018 [32], Uncontrolled before and after study, Australia | First year medical students, Faculty of Monash University | ***mHealth***:  The course included topics on – introduction to course and learning outcomes, computer games for health and wellness, smartphone apps for health and wellness, social networking for health and wellness, 3D applications for health and wellness etc. | Blended Mobile Course (in-class activities including the use mHealth applications to learn about mHealth);  Elective course, Integrated in the first year of medical program | Duration: 10 weeks  Course structure: a hybrid problem-based learning curriculum arranged in themes.  About 300 first-year took 1 elective selected from approximately 20 electives advertised on the Learning Management System (LMS). | Course Development approach: Expert Consultation  Frameworks used: NA | Quizzes (paper-based) | **Attitude** towards the digital health course: increased students’ confidence during the course. |
| Geyer 2008 [56], Uncontrolled before and after study, USA | medical students (unspecified) Albany Medical College | ***Medical informatics:***  The course focused on four areas of medical informatics- (i) Searching Evidence-Based Resources, (ii) Searching Drug Information Resources, (iii) Searching Differential Diagnostic Tools, and (iv) Searching Complementary and Alternative Medicine Resources. | Blended Online Course (Web-based module format, supplemented by classroom or one-to-one teaching sessions);  Elective: Integrated into the medical program | Duration: NA  Course structure:  The first and second years of the LMI curriculum were changed to a Web-based module format supplemented by classroom or one-to-one teaching sessions as needed. Each module includes one or more tutorials and an online exam. All modules are accessible through course management system and remain available to students throughout their medical education. Between years two and three, there is a two-week Clerkship Orientation that prepares students for the clinical environment. | Course Development approach: Evaluation of existing courses; Expert consultation; Piloting the course  Frameworks used: NA | MCQ and short answer questions (online) | NA |
| Gibson 2000 [62], Uncontrolled before and after study, USA | First year medical students, State University of New York | ***Computer literacy:***  the course content included a combination of contents from basic computer literacy course and an introduction to finding medical information on the Internet as well as the campus intranet. | Blended Online Course (Internet search plus hand-on training);  Elective course: "noncredit program" | Duration: 7 hours  Course structure:  the course includes seven hours of hands-on training on computer literacy followed by a test. | Course Development approach: NA  Frameworks used: NA | Survey (online) | **Skills** : the results were improving year on year and students’ success rate correlated positively with entering skill level as measured by the survey. |
| Gjerde 2004 [57], Uncontrolled before and after study, USA | First and Third year medical students, Undergraduate Medical Education for the 21^st^ Century (UME-21) curriculum project taking place in 10 medical schools:  Dartmouth Medical School; University of California, San Francisco; University of Massachusetts;  University of Miami;  University of Nebraska; University of New Mexico;  University of Pennsylvania;  University of Pittsburgh; University of Wisconsin;  Wayne State University | ***Medical Informatics:***  The course consisted of topics related to 5 different types of medical informatic categories such as the role of lifelong learner, role of clinician, role of educator/learner/communicator, role of researcher and role of manager. | Blended Online Course;  Elective course, Integrated into medical school | Duration: The course was taught during the first 3 years of medical school.  Course structure: the course includes demonstrations, lectures, small group tutorials, hands-on labs, task-based assignments, and Internet-based instruction. | Course Development approach: NA  Frameworks used: Association of American Medical Colleges (AAMC) in its Medical School Objectives Project (MSOP) | Survey (online) | **Satisfaction**: Increased satisfaction with the adequacy of training in medical informatics in trained Seniors in UME-21 |
| Gomes 2013 [60], USA, uncontrolled before and after study | Second year medical student; The George Washington University | ***EHR:***  The course covered the contents on the planning, creation, content, and implementation of online educational module on electronic health records. | Online Course (narrated online video of a PowerPoint presentation);  Standalone course | Duration: NA  Course structure: The course consisted of a narrated video that showed PowerPoint slides including explanation on the several functions of the electronic health record system. | Course Development approach: Expert Consultation  Frameworks used: NA | Quizzes, Students’ feedback/comments from the students (online) | **Knowledge**: most students scored highly on the quiz; **Attitude** towards the module: general student feedback was overwhelmingly positive. |
| Jonas 2019 [65], Uncontrolled before and after study, USA | Third year medical students, The Connected Health branch of the Defense Health Agency, the F. Edward Hébert School of Medicine at the Uniformed Services University of the Health Sciences (USU) | ***Telehealth:***  The content covered in the course includes – use of commercial off-the-shelf technologies and telemedicine, current telehealth equipment, mock telehealth encounters via videoconference and preparing medical students to independently deliver telehealth. | Blended Online Course (online plus traditional classroom interaction);  Elective: standalone course | Duration: 9 hours  Course structure: The course was an introductory course on telehealth in the 3^rd^ year and the course included six segments:  (1) multiple-choice pretests;  (2) asynchronous lectures  covering telehealth history, applications, ethics, safety, military uses, etiquette,  and patient considerations;  (3) in-person interactive telehealth instruction including patient selection, current uses, and risk management;  (4) faculty-supervised mock patient telehealth encounters;  (5) hands-on diagnosis and advanced surgical procedures using telehealth equipment; and (6) multiple-choice posttest. | Course Development approach: Expert Consultation  Frameworks used: Training consisted of six segments consistent with Kern’s approach to course development | MCQ (21 items), In person observations | **Knowledge**: Students demonstrated increased overall telehealth knowledge at post-test - 10.1% average improvement between pre- and posttest scores [mean pretest score: 13.76 (SD 1.95); mean posttest score: 15.89 (SD 2.67)] |
| Kern 2011 [4], Case study, Croatia | First and fifth year medical students, University of Zagreb, School of Medicine (Croatia) | ***Medical informatics:***  The course included basic terminology on medical informatics, Windows operating system, text processing, spreadsheet software, presentation software and browsers, e-mail and ethics, Internet, and searching engines. | Mixed: Both Online and Offline Course (Telehealth and offline computer based);  First Year Elective and Fifth Year Mandatory Course, integrated into medical programs | Duration: NA  Course structure: The course presented the approaches to teaching and training medical students in medical informatics at the integrated pre-graduate level: i) basics of medical informatics taught early in the medical curriculum and ii) medical informatics which uses students’ clinical knowledge and is taught towards the end of medical curriculum. | Course Development approach: Literature Review  Frameworks used: IMIA Recommendations on Medical Informatics Education for IT users and adjusted to students’ attitudes to medical informatics issues and the position of the courses in the medical program. | Survey (paper based), seminar presentations | **Attitude** towards MI course: the students reported positive attitude towards MI course |
| Kipnis 2019 [27], Uncontrolled before and after study, USA | First year medical students, Thomas Jefferson University, Academic Information Services and Research | ***Medical informatics***:  The course consisted of topics including searching medical databases, retrieving data from electronic database and other sources, manage and utilization of information for problem solving and decision making. | Online course;  Mandatory course: Integrated into medical program | Duration: 3 weeks  Course structure: The course includes an introductory lecture on medical informatics and online assignments for the studies to complete during the course. To successfully pass the course, the student must complete the two assigned case studies and score 80 points or higher on the post-test. | Course Development approach: NA  Frameworks used: NA | Survey (online) | **Attitude** towards MI course: Student responses indicated that online medical informatics can be successful and the  students had very positive attitudes towards about the online course. |
| Law 2018 [47], Case study, Canada | medical students (unspecified year), University of Toronto | ***Basic programming:***  The course focused mainly on the introduction of programming for medicine. | Blended Offline Course (offline plus traditional methods: computer programming course, homework exercise, seminars);  Standalone a computer programming certificate course for medical students | Duration: 14 months  Course structure: Computing for medicine certificate course includes 3 phases: Phase 1: Introductory (3-4 months) Phase 2: consolidation (5 sessions over a 4-month period) Phase 3: enrichment (6 sessions over a 7 month period) | Course Development approach: NA  Frameworks used: NA | Interviews (qualitative evaluations) | NA |
| Lee 2017 [50], Controlled before and after study, USA | Second and third year medical students; Pritkzer School of Medicine, University of Chicago | ***EHR:***  The course covered the topic mainly on patient-centered EHR use. | Blended Online Course (lectures, online digital);  Integrated into the Clinical Skills course. (The course was formally incorporated into the MS2 Clinical Skills Course). | Duration: 1 hour  Course structure: The lecture on Patient-centered EHR includes a trigger tape video, in which the studies engaged in a reflective observation exercise, and learned best practices (patient-centered communication skills when interacting with electronic health records) | Course Development approach: Expert Consultation  Frameworks used: NA | OSCE, Survey (paper-based) | **Knowledge,:** Compared to third year students, significantly more second year students rated their knowledge (19% vs 55%) and training (14% vs 39%) as good (≥4/5 point scale, P < .001 for both).  **Skills**: Ratings on GOSCE/OSCE performance was higher for the 20 second year students compared to the 88 third year students (73.5 [SD = 4.5] vs 58.1 [SD = 13.1] on 80 point scale, P < .001).  **Attitude** towards EHR training: 39% of second year studies rated ≥4 compared to 14% of third year students. |
| Liaw 2001 [66], Uncontrolled before and after study, Australia | Fifth year medical students; University of Melbourne | ***Computer literacy:***  The course included workshops focusing on the introduction to medical applications and consulting room software including commercial prescribing program. | Blended Offline Course (software, didactic workshops and conversations);  NA | Duration: 1.5 hours  Course structure: The course included the overview lecture and two workshops – i) workshop 1 was conducted in the first week of the 10-week general practice term, and ii) workshop 2 was conducted in the eight week, after the student had experience with diagnostic and management interviews in on-campus tutorials and in general practice placements. | Course Development approach: NA  Frameworks used: NA | Survey (paper-based) | **Skills:** self-reported perceived skill level after the courses – 3.1 to 3.2 out of 5  **Attitude** towards computer use: 82% of students who attended workshop on computer in consultation reported their willingness to use computers in medical practice. |
| Lungeanu 2009 [52], Quasi-RCT, Romania | First year medical students; Victor Babeş University of Medicine and Pharmacy | ***Medical informatics***:  The course covered introductory course on information technology and information skills including topics related to health and medical informatics. | Blended Offline Course: Offline digital (computer) plus homework assignments, in-seminar exercise;  Mandatory course: integrated into medical program | Duration: one semester  Course structure: The course focuses on health and medical informatics including interactive seminar discussions, with practical skills to be acquired as necessary during drop-in sessions in a computer lab. These skills were to be practiced on weekly homework assignments, in-seminar exercises and problem solving; the integration of knowledge and skills was encouraged in formative evaluations spaced throughout the semester. | Course Development approach: Piloting the course  Frameworks used: NA | Surveys,  Open-ended questionnaires (paper-based) | **Satisfaction:** the pilot group felt that the first year HMI course had greater importance for the future and the present [40/55 (73%) of students in the traditional course felt that HMI (in preclinical training) would be important for their future careers] |
| McGlade 2001 [51], Controlled before and after study, UK | First year medical students; The Queen's University of Belfast | ***Medical informatics:***  The contents covered in the course included – managing the data and information in the medical context, communication and the internet, data security and confidentiality, evaluating clinical software and systems, demonstration of clinical systems in hospital and general practice settings, the use of IT and computer in healthcare settings. | Blended Offline Course (lectures, short presentations, practical computer, and field work);  Elective (offered during second semester of first year): integrated into medical program | Duration: The module occupies three 2-hour slots per week over a 12-week period.  Course structure: The medical informatics SSM (special study module) is one of a choice of six electives offered during the second semester of the first year. The course structure includes seminars, demonstrations and practical, fieldwork, assignments, and presentation. | Course Development approach: NA  Frameworks used: NA | Survey (paper-based) | **Skills:** There was a significantly greater use of word-processing (and presentation packages among third-year students compared with second-year students.  **Attitude** toward the course: There was a high level of positive attitude to computers in medicine following the course, in both study and control groups. |
| Mesko 2015 [24], Uncontrolled before and after study, Hungary | Medical and dental students (unspecified years), Semmelweis University, Budapest | ***Computer (Digital) Literacy:***  The contents covered in this course included introduction to social media and medicine, medical search engines and Google applications, online resources in education, the mysteries of medical blogging, using Twitter and other microblogging platforms, medical mobile phone applications, medical aspects of Wikipedia, the future of medicine and social media. | Blended online course (didactic lectures plus online activities);  Elective: integrated into the curriculum | Duration: 12 weeks  Course structure: The elective course is an official part of the medical curriculum  at Semmelweis University, Budapest, Hungary, both in the  Hungarian and English programs. It consists of 10 lectures, each  1.5 hours in length, as well as platforms to openly discussing  the content on the course Facebook page, the written exam, and the 2 online surveys. | Course Development approach: Literature Review  Frameworks used: NA | Written test, survey (online) | **Skills:** The percentage of those responders who  did not know how to define social media decreased from 46.7%  (435/932) to 8.8% (82/932), and those who could define it  perfectly increased from 5.8% (54/932) to 23.2% (216/932)  during the course.  **Attitude** towards the course: 99.7% of the students who participated the course liked the course. Most of the students visited the e-learning platform and found it helpful (74.7%). |
| Milano 2014 [59], Case study, USA | First and Third year medical students; Oregon Health & Science University | ***EHR:***  The topics covered included editing the medication list, updating the  health maintenance tool, placing orders for future studies, referrals, and treatments (based on evidence-based guidelines), the art of triaging care for complex patients, population-based EHR applications, and the professionalism, patient safety, and systems-based care reasons for excellent EHR chart hygiene habits. | Virtual Patient Simulations (EHR simulation);  Mandatory course: Integrated in 3rd year Family Medicine clerkship. | Duration: two weeks.  Course structure: EHR Course includes hands-on activities in a simulated EHR using a virtual patient. Small groups of six to eight students meet with a faculty facilitator during the second weeks of the clerkship for two-hour session to discuss the first week's work and the content of the "correct" chart (patient problem list, allergies, and history sections). | Course Development approach: Literature Review, Expert Consultation; Piloting the course  Frameworks used: NA | In person observation, rating, surveys (both online and paper-based) | **Students’ Skills**: modest improvement in the more experienced students’ ability to place orders and update the chart in EHR, including the allergy section and medication list, when comparing data for students who completed the Sim-EHR exercise early versus later in their third year. |
| Pereira 2018 [61], Uncontrolled before and after study, USA | Medical students (third and fourth year) | ***EHR:***  The topics covered in the course includes – writing notes from scratch, using chart review, navigating results review, writing notes using smart phrases, finding your patient, reviewing the problem list, entering orders, using order sets, performing medication reconciliation, customizing a patient list, writing notes in NoteWriter, and using admission/discharge navigator. | Online Course;  Integrated into third year medical program | Duration: NA  Course structure: EHR course covers topics on  i) Epic environment overview,  ii) order entry,  iii) patient information review  iv) chart documentation | Course Development approach: Expert Consultation  Frameworks used: NA | MCQs (online) | NA |
| Schwartz 2011 [58], Uncontrolled before and after study, USA | First year medical students; Rocky Vista University College of Osteopathic Medicine | ***Medical informatics:***  The initial lecture covered the classification and location of information. The second lecture provided instruction in verifying the quality, accuracy, and timeliness of information and the final lecture culminated with strategies for presenting retrieved information in a variety of settings and formats. | Blended Offline Course (PowerPoint plus lecture plus in person training);  Mandatory: integrated into medical program, a year long, for-credit program | Duration: One year  Course structure: the course consists of three lectures  delivered by the informatics faculty. Learning activities included didactic lectures, assigned readings, and ultimately a formal public case presentation. Course was designed toward preparing students to fulfill the final requirement, the case presentation, as an indicator that they had acquired and honed information literacy skills. | Course Development approach: Expert Consultation  Frameworks used: NA | Quizzes, Essays (writing exercise), Surveys | NA |
| Seago 2008 [48], Case study, USA | Medical students (first, second and third year) | ***Medical informatics:***  The course covered different topics related to medical informatics including hospital information system, computer-based instruction and instructional software, personal digital assistant in clinical practice and the use of electronic mail. | Mixed: Online and Offline Courses;  Elective: Integrated into medical program | Duration: throughout the first three years of medical school.  Course structure: the course extended across 11 first-year courses, 15 second-year and 7 clinical clerkships during the third year of medical program. All syllabi for first- and second-year courses are Web-Based and produced by the school of medicine curriculum office and are available on the eCurriculum Web site. | Course Development approach: NA  Frameworks used: NA | NA | NA |
| Silverman 2012 [28], Uncontrolled before and after study, USA | First to fourth year medical students; University of Arizona College of Medicine | ***Biomedical informatics:***  The course focused on an overview of  BMI, privacy and security issues, and the use of online and handheld clinical decision support calculators and tools.  The course also covered the topics on data, decision making and safety issues. Additionally, the longitudinal themes threaded across all integrated system blocks including behavior science, biomedical informatics, medicine and society, public health, population medicine and prevention. | Blended Offline Course (used TreeAge software) plus lectures and lab;  Mandatory course - first to third year  Elective module – fourth year, Integrated in the first and second year of medical program | Duration: one-month  Course structure: The course extends across the first three years of the medical school curriculum. A 1-week, second-year BMI block involved 19 hours of instruction in decision making, decision analysis, and clinical  decision support. | Course Development approach: Expert Consultation, Piloting the course  Frameworks used: NA | Surveys (paper-based) | **Knowledge:** Self-  assessment of BMI knowledge increased  after exposure to the course. Students attending the BMI course  were able to answer objective  questions related to BMI content. |
| Wagner 2010 [29], Uncontrolled before and after study, USA | Medical students (preclinical years) | ***EHR:***  The course presented the topic on the development and evaluation of Health Record Online Submission Tool with the features of health record tracking and organization of submissions and automated notification systems. | Blended Online Course (online lectures, a written tutorial);  Mandatory: Integrated into medical program | Duration: throughout the preclinical medical program  Course structure: The course included several domains throughout preclinical years including:  - Lectures  - A written tutorial  - Writing of 17 graded health records | Course Development approach: NA  Frameworks used: NA | Rating (online) | **Attitude** towards the course**:** no difference before and after the course on "Health Record Online Submission Tool (HOST)"  **Faculty's attitude** towards the course: it saved time and made the grading simpler |
| Wald 2014 [30], USA, Uncontrolled before and after study | Medical students (third and fourth year) | ***EHR:***  The course included 4 different subtopics - i) introducing students to the presence of a computer within a clinical encounter, ii) training students in EHR-related skills, iii) empowering patient- and relationship-centered interviewing skills while incorporating EHR skills, and iv) fostering students’ appreciation for added value of integrated computer use within the clinical encounter". | Blended offline course (Didactic PPT plus EMR training);  Elective course: Integrated into medical program | Duration: throughout third and fourth year of medical school  Course structure: The course described the process of bridging theory into practice with systematic  longitudinal curriculum development for EHR training in undergraduate medical education based on Kern and colleagues’  curriculum development framework. The authors used reflective learning and practice as foundation competency for designing EHR course for third- and fourth-year medical students. | Course Development approach: NA  Frameworks used: NA | Multisource feedback for students, including (1) reflective self-assessment, given the potential benefit of making specific criteria available to guide self-directed reflective learning, and (2) feedback within practice sessions from multidisciplinary faculty and standardized patients. | NA |
| Wan 2016 [63], Case study, Taiwan | medical students (Unspecified), Taipei Medical University | ***Computer literacy:***  The course covered topics including  introduction to hardware, software, Internet, information security  and privacy, copyright, and creative commons, and related applications. | Blended Online Course (online game and book club);  Standalone - "an entry-level election for undergraduate in a medical university" | Duration: 17 weeks (2 hours/week)  Course structure: Basic Computer Concept (BCC) course, which is an entry-level election for undergraduate in a medical university. The course included 3 different components:  a) self-learning from Open Education Resources  ii) Cooperative learning from a Book club.  iii) Game-learning from an online Jeopardy-like game | Course Development approach: NA  Frameworks used: NA | (1) survey and open questionnaires,  (2) ratings  (3) assignments (grading)  (4) system-based metrics (user clicks, reading time and discussion rate) (both computer-based offline and paper-based) | **Skills:** students who spent more time on the learning  content generally scored higher in grades and there is a positive correlation between the final grades and the reading records.  **Engagement**: Students who spent more time on the learning content generally scored higher in grades (i.e. 65% of the students claimed that they had read  more than 75% of Open Educational Resource content.) |

Abbreviations: AAMC - Association of American Medical Colleges; ACGME- Accreditation Council for Graduate Medical Education; BMI – Biomedical Informatics; EHR – Electronic Health Record; HMI – Health and Medical Informatics; IT – Information Technology; LMS - Learning Management System; Moodle - Modular Object-Oriented Dynamic Learning Environment; NA – Not Available ;OSCE – Objective Structured Clinical Examination; SD – Standard Deviation; UME -21 - Undergraduate Medical Education for the 21^st^ Century; USA – United States of America; UK – United Kingdom; * Detailed of the outcome results were not presente
