# Supplementary figures and images for: Digital Health Training Programs for Medical Students: Scoping Review
Source: JMIR Med Educ. 2021 Jul 21;7(3):e28275. doi: 10.2196/28275 (PMC8339984; doi:10.2196/28275)

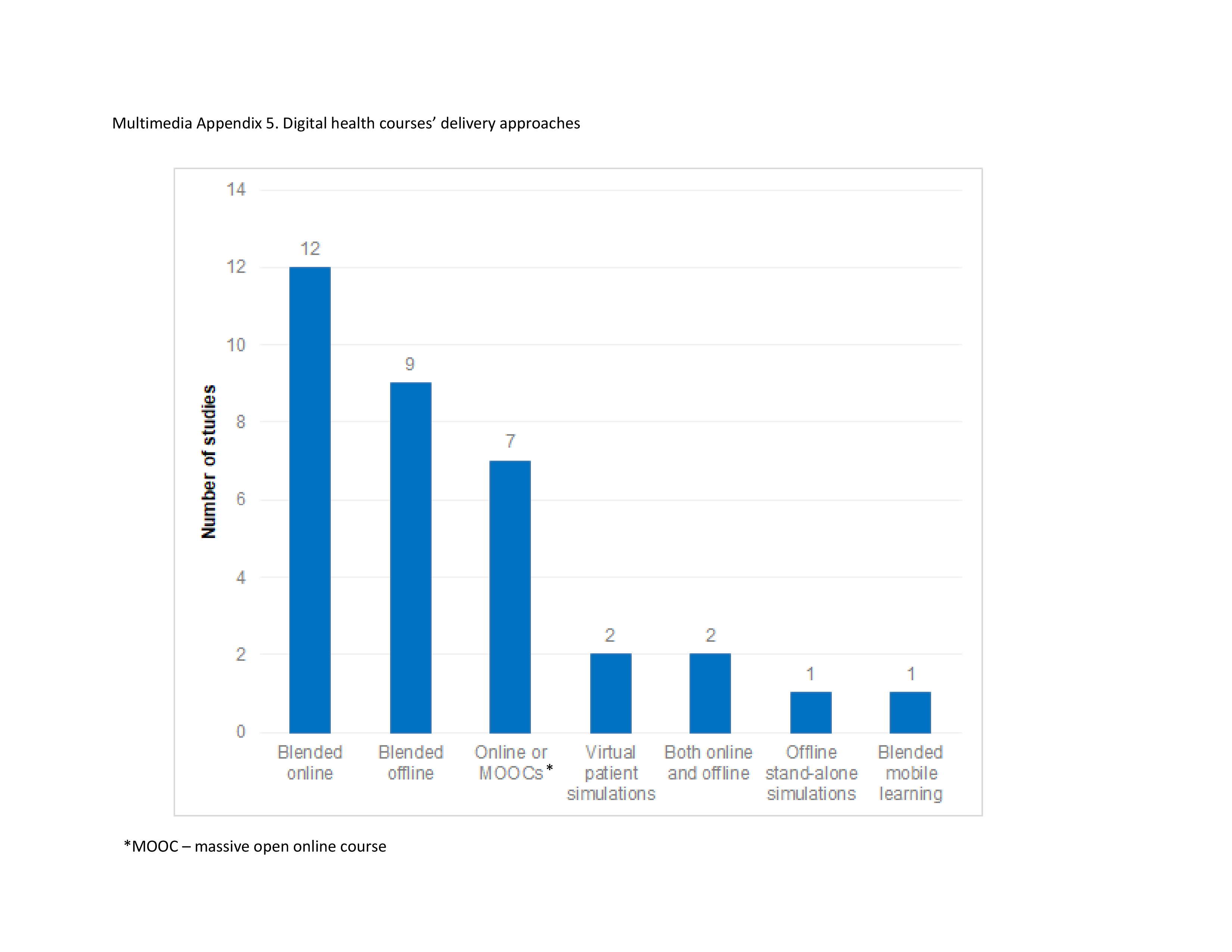

Supplement: Multimedia Appendix 5 [file mededu_v7i3e28275_app5.png]
