# Supplementary material for: Digital Health Training Programs for Medical Students: Scoping Review
Source: JMIR Med Educ. 2021 Jul 21;7(3):e28275. doi: 10.2196/28275 (PMC8339984; doi:10.2196/28275)
Supplement: Multimedia Appendix 6 [file mededu_v7i3e28275_app6.docx]

**Multimedia Appendix 6.** Digital health course development process and learning objectives.

| **Study ID** | **Digital health category (e.g. telemedicine, mHealth, EHR, etc.)** | **Course/Curriculum development process** | **Learning objectives of the course/curriculum** | **Learning objectives - how were they developed?** |
| --- | --- | --- | --- | --- |
| Behrends 2011 [53] | Medical informatics | Not available | The course introduced medical students to the basics of medical informatics (i.e. clinical decision support systems, medical information systems, healthcare telematics, data privacy protection, health technologies, medical image processing, biosignal analysis as well as general aspects of using the internet in medicine). | Not available |
| Blumenthal 2005 [18] | Medical informatics | **Literature Review:** conducted by interdepartmental informatics curriculum committee (including librarians, faculty members, and medical students) reviewed the informatics objectives included in the Association of American Medical Colleges Medical School Objectives Project and the general competencies included in the Accreditation Council for Graduate Medical Education Outcomes Project.   **Survey:** A structured survey completed by Georgetown University School of Medicine course and clerkship directors identified the informatics instruction in numerous preclinical and clinical courses but no coordination of content or overall picture of the knowledge and skills needed by aspiring physicians. | **The learning objectives for the preclinical Informatics course were**: • Demonstrate the ability to retrieve, manage, and apply information from electronic sources to address public health care issues • Appreciate the challenges in obtaining county-level data   **The learning objectives for the clinical Informatics course were**: • Develop a foreground question based on a real patient encounter  • Translate the question into a searchable, “answerable” one using PICO (i.e. Population, Intervention, Comparison, Outcomes) • Identify knowledge gaps or related background questions  • Use Ovid (MEDLINE and EBM Reviews) and at least one other clinical resource to answer the questions. | **Preclinical Informatics** Evaluation on the medical informatic course and assignments by the course director, project director, and librarian   **Clinical Informatics** Not available |
| Breil 2010 [54] | Medical Informatics | Included 3 steps:  (1) **Evaluation of existing course**: the authors carried out the standard evaluation of an existing course in informatics for medical students during the term 2007/2008 to summer term 2009. (2) **Piloting the course:** The authors incorporated experiences from a pilot course on management of medical information systems which was introduced in the previous year.  (3) **Literature Review:** The authors analyzed literature concerning medical informatics education with a focus on multidisciplinary courses | The course aimed to integrate the management of information system in medical education and to provide medical students with practical experience related to the design and implementation of medical information system. | Not available |
| Brockes 2017 [45] | Telemedicine | Not available | The course learning objectives were to:   - enable the students to describe the basic principles of telemedicine, specifics of the telemedical consultation, including legal aspects, data protection, and potentials, as well as limits and sources of errors. - To enable the students to judge important symptoms of diseases, when the patient is separated from the doctor, to match the symptoms to a disease, as well as to create an appropriate consultation for the patients. | Not available |
| Brown 2003 [25] | Medical informatics | Included 2 steps:  (1) **Curriculum Review:**  Based on curriculum Revision Committee, which included a librarian, the chairs of each of the revision committees, members of the Coordinating Committee that oversaw the process.  (2) **Expert consultations**:  Librarians and experts in the curriculum review committee provided inputs for curriculum design and development | The course learning objectives were 1. To Be able to formulate a searchable question from a clinical scenario, to translate a question into a search strategy.  2. To conduct an efficient and competent MEDLINE search, and to select relevant articles to answer a specific question" | Learning objectives were based on Association of American Medical Colleges (AAMC)'s medical objectives |
| Bulik 2010 [64] | Telemedicine | Not available | The objectives of the course were to expose medical students to telemedicine as a method for delivery of primary care and to reinforce the importance of doctor-patient communication during the health-care encounter. | Not available |
| Burgun 2006 [49] | Medical informatics | Not available | Learning objectives of the course were to:  1. Give a precise deﬁnition of the topics on which you are searching for information, e.g., disease description, prognosis, evolution, treatment, and biological mechanisms involved.  2. Deﬁne the concepts of resource, site, document, web page.  3. List and categorize the tools that are available: search engines, specialized search engines, portals and catalogs, sites that produce information.  4. Describe and explain how these tools work (data vs. metadata, automatic vs. manual indexing, thesaurus vs. natural language, database vs. web pages).  5. Deﬁne precision and recall in information retrieval and apply it.  6. Explore and test decision support systems accessible on the internet and provide comments.  7. Categorize resources according to their purposes and their targets (educational resources, professional, research, consumer health).  8, Categorize resources according to the editor (universities, patient associations, US National Library of Medicine, etc.).  9. Clarify the notion of URL, domain name and use them as a criterion for resource categorization (.com, .edu, .gov, .fr, .org, etc.)  10. Formulate problems about the quality of information in the internet.  11. Enumerate the 10 more important quality criteria  12. Apply quality criteria to a given internet resource  13. Build strategies for collecting relevant information via the internet  14. Evaluate the documents that are accessible on the web and compare them to reference (paper textbooks, etc.) | Not available |
| Burnette 2012 [26] | Medical informatics | **(1) Expert consultations**: librarians collaborated with medical faculty during the second-year introduction to clinical medicine course to lead small group learning in the use of evidence-based resources to answer case-based questions. The subcommittee also recommended developing a medical informatics elective that would enhance existing informatics instruction and better prepare students for residency, clinical practice, and lifelong learning.   **(2) Literature review**: A search for other models of medical informatics instruction revealed that the elective format was not without precedent.  The five future roles for medical students (clinician, researcher, manager, educator-communicator, and lifelong learner) identified in the Medical School Objectives Project provided the initial organizational framework | to the learning objectives for the course::  1. understand what medical informatics encompasses and how it relates to various roles in the practice of medicine, including clinical care, research, teaching, and lifelong learning 2. retrieve, evaluate, and apply medical information as it pertains to each of the major four roles addressed in the course 3. consider the basics of electronic medical records, patient management systems, decision support tools, patient safety, and patient education as they apply to clinical care and practice management  4. develop a personal information management plan | -Based on an overview of MI focusing on basic principles, concepts, technology, and terminology.  -An inventory of informatics topics and research skills already placed across the curriculum served as the foundation for new or supplemental content for the elective.  -The five future roles for medical students (clinician, researcher, manager, educator-communicator, and lifelong learner) identified in the Medical School Objectives Project provided the initial organizational framework. |
| Connor 2003 [55] | Medical informatics | Not available | The learning objectives for students include developing skills related to communicating, critical thinking, and problem solving; using information technology; managing large volumes of knowledge; using clinical practice guidelines; and valuing lifelong learning. | Not available |
| Connors 2007 [19] | EHR | Not available | The course learning outcomes were enabling students to: • document the consult electronically,  • use decision-making tools, • develop critical thinking skills, • make decisions with data, • understand the importance of structured nomenclature, • acquire information at point of learning, • search databases, and • access evidence-based guidelines with supporting reference text.. | Not available |
| Ferenchick 2013 [3] | EHR | Not available | The course aimed to access meaningful use (MU) of electronic medical records and the performance of medical students on electronic analysis of data entered into structured fields using decision support tools as well as the correlation between the students’ MU performance and other measures of educational attainment. | Not available |
| Fernandez-Marcelo 2012 [46] | Medical informatics | **Expert Consultation:** University of Phillippine (UP) medical students (and resident physicians) learn to use another electronic medical records system, the Integrated Surgical Information System or Integrated Surgical Information System, for hospitalized patients of the Department of Surgery, UP-Philippine General Hospital. This was also built using free and open source tools by the Department of Surgery and the MIU. ISIS was embedded into the academic clinical rotations in Surgery for four and eight weeks during their senior and internship years, respectively." | The course aimed to enable the students to use medical informatics systems including electronic medical records system and surgical information system. | Not available |
| Fernando 2018 [32] | mHealth | **Expert consultation:** the Australasian College of Health Informatics membership made several suggestions for meaningful Learning outcomes on their e-mail forum. Members often used their own professional networks to support design and development of the elective. | The course learning objectives were to enable students to 1. Articulate some differences between the application of games, social media, smart- phone applications on mHealth enabled devices for health and wellness 2. Assess many new and emerging e-tools for health and wellness 3. Assess many risks and benefits that games, social media, and smart-phone applications present in the context of health promotion and own learning and practice skills 4. Navigate to and search for the m-health tools and install them on devices as required.  5. Develop basic research and presentation skills | Learning outcomes (LOs) for the mHealth component were developed based on the feedback from students, consultation with colleagues, familiarity with the relevant educational and health informatics literature, medical registration and regulatory expertise, and professional competence. |
| Geyer 2008 [56] | Medical informatics | **(1) Expert consultations, (2) Curriculum Review and (3) Piloting the course:** The process of curriculum review was carried out in 2003 - Anecdotal and quantitative data gathered over the past years, including exam results, course evaluations, and faculty feedback were reviewed for trends and opportunities for improvement. Recognizing that the timing and placement of LMI (LeGrange Medical Informatics) content was predominantly isolated from themes and clerkships, the informatics curriculum was changed to teach knowledge-based tools and technologies as they fit naturally into the basic science and clinical courses.  The study also used planning protocol to assist faculty in developing modules. It defines a six-step process that begins with articulating outcome-based course objectives and ends with outcome measurement tools.  **Course/Class Planning Protocol**  Step 1: Articulate goals, objectives, & content  Step 2: Define measurable objectives  Step 3: Map content to objectives  Step 4: Select method(s) of delivery; describe learning environment  Step 5: Create a strategy for the assessment(s) and evaluation  Step 6: Determine appropriate assessment instruments for each objective | The course aimed to enable students to use available information resources to conduct research, locate medical/health information on the Web using Google, and conduct a literature search on MEDLINE using PubMed’s interface.  In year two, the course aimed to enable students to equip with to locate complementary and natural medicines information, use evidence-based medicine searching techniques, retrieve drug information using available databases, and use differential diagnostic tools to assist in diagnosing a disease. | Based on the following steps: Step 1: Articulate goals, objectives, & content  Step 2: Define measurable objectives  Step 3: Map content to objectives  Step 4: Select method(s) of delivery; describe learning environment Step 5: Create a strategy for the assessment(s) and evaluation Step 6: Determine appropriate assessment instruments for each objective |
| Gibson 2000 [62] | Computer literacy | Not available | The course learning objectives were to enable students to :  *General*   - Create an organized structure of personal directories or folders  - Create a new document and save it to a disk using the directory structure  - Be familiar with basic formatting options in a word processor  - Cut and paste data between documents created by the same or different applications  - Log-in to a file server and download a document or application using file sharing or file transfer protocol *E-mail* - Log into an e-mail account  - Create and send e-mail  - Receive and read an e-mail and save a message to a folder  - Attach a file to an e-mail  - Create a personal signature  - Subscribe to an electronic mailing list *Internet* - Access files on the Internet via direct input of addresses, hyperlinks, and use of search tools  - Create bookmarks and bookmark folders  - Save the contents of a Web page as a text file  - Utilized advanced search engine syntax to limit retrieval to relevant Web sites *Networked resources* - Register to use the library’s online bibliographic databases (MEDLINE, EMBASE, etc.)  - Save the results of a MEDLINE search and the strategy by download or e-mail  - Use Medical Subject Headings (MeSH), logical operators, and limits to create successful search strategies  - Search the library’s online catalogue and complete an author, title, journal title, and subject search | Planning and discussion in advance by the dean of medicine and curriculum subcommittee to introduce a set of concrete goals that encompassed understanding basic concepts as well as the ability to perform specific task related to computer literacy competencies |
| Gjerde 2004 [57] | Medical Informatics | Not available | The course aimed to equip students with the competencies related to the Medical informatics literacy categories as described in the MSOP (Medical School Objectives Project) : role of the lifelong learner, role of clinician, role of educator/learner/communicator, role of researcher/evaluator, and role of health care system manager. | Not available |
| Gomes 2013 [60] | EHR | **Expert consultation**: including EHR vendors, library directors | The course aimed to provide students with basic information about the history, challenges, and use of electronic health records. | Not available |
| Jonas 2019 [65] | Telehealth | **Expert consultation:** Nine-hour course was developed in close partnership with telehealth educational design and curriculum development experts at the Connected Health branch of the Defence Health Agency. An interprofessional panel of telehealth experts selected and created asynchronous materials deemed essential for conducting a telehealth encounter. | The course objectives were to (1) use commercial off-the-shelf and military-specific technologies to teach telehealth, (2) expose students to current military telehealth equipment and applications, (3) practice faculty-supervised mock telehealth encounters via videoconference, (4) leverage technology to teach and prepare medical students to independently deliver telehealth, and (5) respond to calls by the AMA and the NDAA to improve provider comfort and proficiency with telehealth. | Not available |
| Kern 2011 [4] | Medical informatics | **Literature Review:** Both Basics of medical informatics and Medical informatics are courses tailored in line with the IMIA Recommendations on Medical Informatics Education for IT users, and adjusted to students’ attitudes to medical informatics issues and the position of the courses in the medical curriculum. | The course aimed to enable students to skilfully and competently use ICT, through practical in the computer laboratory.  The specific course learning objectives were: (1) to introduce the students to the concept of MI, as well as develop their skills in working with health data relevant for practice and research in medicine and health care (coding, structuring, databases);  (2) to increase awareness of standards, classifications, data protection, and system security, as well as ethics in the use of ICT;  (3) to introduce methods of knowledge discovery as well as the concept ICT-based support for medical decision making;  (4) to provide insight into real ICT applications for assessing their appropriateness/ usefulness for a practicing physician;  (5) to develop students’ skills needed for searching bibliographic databases and other Internet based sources of medical knowledge | Not available |
| Kipnis 2019 [27] | Medical informatics | Not available | The course was designed to demonstrate the need for lifelong-learning skills, to train students in how to ask the appropriate questions to find an answer to their information needs, and to instill an awareness of the various types of information sources available to them and the skills to use these resources. | NA |
| Law 2018 [47] | Basic programming | Not available | Objectives of the course were as follows:  **Phase 1**: The objectives of this phase were to enable students to write very basic Python programs; trace basic Python programs involving lists, dictionaries, and files; and recognize good practices in software design. **Phase 2**: Focus on consolidation of learning over time -The objectives of this phase were to enable students to write programs that combined concepts from phase I, write programs to solve a problem, use good practice in software design consistently, and use a debugger to find mistakes in a program written by another author. **Phase 3:** To enhance their understanding of how computing can be applied to medicine through six 2-hours seminars. | Not available |
| Lee 2017 [50] | EHR | **Expert Consultation:** To provide students with a high-fidelity outpatient scenario, investigators worked with the University of Chicago’s EHR trainers to design a mock-chart in the training environment with five years of clinic notes and studies. During the GOSCE/OSCE, students logged into the mock chart, addressed the standardized patient’s (SP) chief concern, reviewed the EHR for relevant clinical information (i.e., labs, notes), and provided appropriate counselling. | The objectives of the courses were to:  (1) highlight benefits of EHR use in clinical care  (2) identify barriers to patient-centred EHR communication  (3) introduce best practices and  (4) allow for skills practice and feedback. | Not available |
| Liaw 2001 [66] | Computer literacy | Not available | The course aimed to enable the students to: 1) improve understanding of current clinical informatics resources and their use; 2) improve skill and confidence in the use of clinical applications, and  3) improve the consulting skills required when using computers in the consultation. | Not available |
| Lungeanu 2009 [52] | Medical informatics | **Piloting the course:** Interactive teaching approach - started a pilot course on HMI (Health and Medical Informatics) knowledge in interactive seminar discussions, with practical skills to be acquired as necessary during drop-in sessions in a computer lab aimed at testing an explicitly interactive approach to teaching HMI, with a formative evaluation procedure for students’ knowledge. These skills were to be practised on weekly homework assignments, in-seminar exercises and problem solving; the integration of knowledge and skills was encouraged in formative evaluations spaced throughout the semester. | The course aimed to enable the students to critically evaluate each ICT tools and use them responsibly and instil life-long learning styles | Not available |
| McGlade 2001 [51] | Medical informatics | Not available | The course aimed to improve medical students' use of computer technology and IT skills | Not available |
| Mesko 2015 [24] | Computer (Digital) Literacy | **Literature Review:** based on social media use trends | The course aimed to teach students how to use the Internet, with a special emphasis on social media. | Not available |
| Milano 2014 [59] | EHR | **(1) Literature Review, (2) Expert Consultation and (3) Piloting the course**  Included three development steps: 1. Identified EHR-related general and specific objectives for the curriculum using existing guidelines and input from OHSU faculty members responsible for EHR implementation  2. Designed the objectives to impart general concepts common to all EHRs rather than related to a specific EHR platform.  3. Created a virtual patient and a simulated chart in which trainees can practice these skills. | The course aimed to enable students to maintain an accurate EHR documentation (correct/update social, surgical, and family histories, allergy alerts, and problem and medication lists), apply prevention and chronic disease guidelines in the EHR context, and order appropriate referrals/consultations, laboratory tests, imaging studies, and medications. | Based on existing guidelines and input from faculty members responsible for EHR implementation |
| Prereira 2018 [61] | EHR | **Expert Consultation:** Identified the problems/needs assessment from the lead Epic educators responsible for training at the six health systems that use Epic in Minnesota. Delphi survey was used to select essential topics for the course. | The course learning objectives were to develop the following skill in students:   - writing notes from scratch - Using chart review - Navigating results review - Writing notes using SmartPhrases - Finding your patient - Reviewing the problem list - Entering orders - Using order sets - Performing medication reconciliation - Customizing a patient list - Writing notes in NoteWriter - Using admission/discharge navigator | Based on the feedback from Epic educators, the essential components for a course were selected. The essential topics were then refined via a Delphi process. |
| Schwartz 2011 [58] | Medical informatics | **Expert Consultation:** The librarians/faculty members teach courses on critically appraising resources. With input from the clinical faculty and bench researchers, the librarians began to put together the structure of the medical informatics course, meeting monthly for a year to develop objectives, learning activities, and assessment tools. | The course learning objectives were to enable students to: to:  (1) answer basic questions about information, information systems, and the role of information literacy and informatics in health care  (2) recognize the need for case-specific information and articulate a well-formed question  (3) develop effective search strategies and apply them to selected databases  (4) evaluate and synthesize information retrieved in a search  (5) adhere to ethical and professional standards of information use  (6) design and deliver an informed presentation of a clinical case. | Developed by Discussion through faculty and administration |
| Seago 2008 [48] | Medical informatics | Not available | The course aimed to enable development of competencies among students relating to five physician roles as outlined in the Medical School Objectives Project (MSOP) by the The Association of American Medical Colleges (AAMC): the lifelong learner, clinician, educator/communicator, researcher, and  manager. | The Association of American Medical Colleges (AAMC) published the medical informatics objectives as part of its Medical School Objectives Project in June 1998 and served as guidelines for the teaching of informatics within the medical school curriculum. |
| Silverman 2012 [28] | Biomedical informatics | **Piloting the course:** the authors developed a 1-week, 13-hour, first-year Biomedical Informatic (BMI) block to create the medical school campus in Phoenix (COM-P) curriculum. Initially, this block was focused on an overview of BMI, privacy and security issues, and the use of online and handheld clinical decision support calculators and tools. A 1-week, second-year BMI block involved 19 hours of instruction in decision making, decision analysis, and clinical decision support. This block was taught in lectures and labs in which student groups developed decision trees using TreeAge Pro decision analysis software. | The course learning objectives were are follows: **For Day One:**  1) Define database and describe its component parts.  2) Define data schema and describe its component parts.  3) Describe the different data types and explain their uses.  4) Create a data table from a data schema and vice versa.  5) Describe historical examples of studies using biomedical informatics techniques which changed medical practice.  6) Review the prevalence, symptoms, etiology, diagnosis, treatment, and prognosis of Huntington disease.  7) Discuss the organization and utilization of data to address key questions related to genetic disorders such as Huntington disease.  8) Create data schemas for clinical, genetic, and population data related to Huntington disease.   **For Day Three**:  1) Discuss the definition of the terms database, table, column, and row.  2) Review the definition of data granularity and its impact on merging data sets.  3) Describe strategies for resolving naming, data granularity, and data compositional issues when merging data sets.  4) Demonstrate the ability to link data across domains (gene, patient, population) to answer meaningful translational (cross-domain) questions for a given condition,  5) Describe examples of two “real-world” data integration examples and comment on the challenges and opportunities of the data integration in these examples. | Based on BMI educational objectives from the Association of American **Medical Colleges Medical School Objectives Project (MSOP), learning objectives were developed.** Information literacy learning objectives were compiled as a separate list and were coordinated and taught by a team of faculty with expertise in BMI, public health, and library sciences.   Second, each learning objective was mapped to be taught in one or more of the specific curricular elements (mapped learning objectives versus key themes the course). |
| Wagner 2010 [29] | EHR | Not available | The course aimed to enable the students to prepare for the use of electronic health records | Not available |
| Wald 2014 [30] | EHR | Not available | The objectives of the course were:  1) introducing students to the presence of a computer within a clinical encounter  2) training students in EHR-related skills,  3) empowering patient- and relationship-centred interviewing skills while incorporating EHR skills, and  4) fostering students’ appreciation for added value of integrated computer use within the clinical encounter" | Not available |
| Wan 2016 [63] | Computer literacy | Not available | The objectives of this introductory course were to teach students:  (1) how to use IT tools to present their ideas,  (2) where to find answers or resources of their homework or research through Internet or databases, and  (3) how to cooperate with each other through internet. | Not available |

Abbreviations: EBM - Evidence Based Medicine; EHR – Electronic Health Record; EMBASE - Excerpta Medica database; GOSCE - Group Objective Structure Clinical Examination; ICT - Information and Communication Technologies; IT – Information Technology; IMIA - International Medical Informatics Association; MEDLINE - Medical Literature Analysis and Retrieval System Online; MI – Medical Informatics; OSCE - Group Objective Structure Clinical Examination
